# Extracellular vesicle-mediated signalling in human rescue *in vitro* maturation: methodological and mechanistic considerations

**DOI:** 10.1093/hropen/hoag049

**Published:** 2026-05-24

**Authors:** Xingyu Sun

**Affiliations:** Department of Gynecology, The Affiliated Traditional Chinese Medicine Hospital, Southwest Medical University, Luzhou, Sichuan, China

Dear Editor,

I read with great interest the recent article published in *Human Reproduction Open* by Makieva *et al*. (2026) that described the beneficial effects of follicular fluid–derived extracellular vesicles (ffEVs) on human rescue *in vitro* maturation (rIVM). The authors demonstrate that supplementation with ffEVs significantly increases the proportion of oocytes reaching metaphase II (MII) and is associated with changes in oocyte ultrastructure and proteomic profiles. These findings provide intriguing evidence that extracellular vesicle (EV)-mediated communication within the follicular microenvironment may influence oocyte maturation and potentially improve outcomes of assisted reproductive technologies. While the study offers valuable insights, several methodological and interpretative aspects warrant further discussion.

First, the extracellular vesicle preparations used by Makieva *et al*. (2026) were obtained using differential ultracentrifugation, a commonly used approach that enriches vesicles but may also co-isolate non-vesicular components, including protein aggregates and lipoproteins. Although particle characterization and vesicle markers were reported, ultracentrifugation-derived fractions are generally regarded as EV-enriched preparations rather than highly purified vesicles (Welsh *et al*., 2024). Consequently, it remains difficult to exclude the possibility that some of the observed biological effects may be partially attributable to soluble follicular fluid components that co-sediment during ultracentrifugation. Complementary isolation strategies or functional depletion approaches could help clarify whether the observed maturation effects are specifically mediated by vesicular cargo.

Second, Makieva *et al*. (2026) employed pooled follicular fluid samples from multiple donors to generate ffEV preparations. While this strategy improves experimental feasibility and reduces technical variability, it may also mask biologically relevant heterogeneity among patients. Emerging evidence suggests that the molecular composition of follicular fluid extracellular vesicles varies across patient subgroups, including differences in reproductive age and metabolic status, and such inter-individual variability may influence vesicle cargo and biological activity (Sysoeva *et al*., 2024; Chang *et al*., 2025). Therefore, evaluating donor-specific or follicle-specific EV populations may provide additional insights into the variability and reproducibility of EV-mediated effects on oocyte maturation.

Another point that deserves attention relates to the interpretation of maturation outcomes. The primary endpoint of Makieva *et al*. (2026) was polar body extrusion, which reflects nuclear maturation. However, nuclear maturation alone does not necessarily indicate improved developmental competence. Cytoplasmic maturation, mitochondrial function, chromosomal integrity, fertilization potential, and embryo developmental capacity represent additional key determinants of oocyte quality (Baldini *et al.*, 2024). Thus, although the increase in MII oocytes observed in the ffEV-treated group is encouraging, further studies assessing fertilization rates, embryo development, and genomic stability will be necessary to determine whether EV supplementation truly enhances developmental potential.

The proteomic observations reported by Makieva *et al*. (2026) also raise interesting mechanistic questions. Notably, hyaluronan synthase 1 (HAS1) was among the most significantly upregulated proteins in ffEV-treated oocytes, despite being absent from the vesicle proteome itself. This finding suggests that the observed changes may not result from the simple transfer of vesicular proteins but rather from secondary signalling responses within the oocyte. Increasing evidence indicates that extracellular vesicles can deliver regulatory RNAs and signalling molecules capable of reprogramming recipient cell transcriptional and metabolic pathways (Cerrotti *et al*., 2024). In this context, vesicle-mediated modulation of pathways related to mitochondrial activity, lipid metabolism, or endoplasmic reticulum organization may contribute to the structural changes observed in Makieva *et al*. (2026).

Further characterization of the transcriptomic and small RNA cargo of follicular EVs, together with functional validation of candidate signalling pathways, may therefore be crucial for elucidating the molecular mechanisms underlying EV-mediated enhancement of oocyte maturation. Such mechanistic insights would also be important for developing standardized EV-based supplementation strategies for IVM systems.

In conclusion, the study by Makieva *et al*. (2026) provides an important step towards understanding the role of EV-mediated signaling in the follicular microenvironment. At the same time, additional mechanistic and functional investigations will be required to clarify the precise drivers of the observed effects and to determine whether ffEV supplementation ultimately translates into improved developmental competence and reproductive outcomes.
